# Construct validity of instrumented gait assessments in hospital and daily life mobility in patients with Parkinson’s disease and atypical Parkinson's syndromes: an exploratory study

**DOI:** 10.1007/s00415-026-13652-0

**Published:** 2026-02-09

**Authors:** I. Teckenburg, V. Sidoroff, H. Moradi, S. Sapienza, G. Prigent, F. Krismer, D. Benninger, B. Eskofier, M. Regensburger, A. Ibrahim, J. Klucken, K. Aminian, G. Wenning, C. Raccagni, J. Winkler, H. Gaßner, Mobility_APP Study Group, Patrick Bachmann, Georg Göbel, Helene Hummer, Frank Jagusch, Jean-Pierre Ndayisaba, Gudrun Schoenherr, Stefan Kiechl, Werner Poewe, Johanna Wüstner, Anna Resch, Svenja Schmidt, Pascalle Hendriks, Raphaela Greimann, Hannah Huber, Klaus Seppi, Kathrin Kinscher, Nina Hergenröder-Lenzner, Jelena Jukic, Sabine Stallforth, Alexander German, Christina Erhardt, Patrick Süß, Emily Adler, Andrea Dötzer, Ilaria Scarano, Michaela Peranzoni, Barbara Zaniboni, Karin Voelkl, Martina Bortolini, Anisoara Paraschiv-Ionescu, Marzieh Asalian, Nils Roth, Lisann Lieb, Felix Kluge, Marijus Giraitis, Gelani Zelimkhanov, Olena Tsurkalenko, Eva-Tabea Schoenfeldt-Reichmann

**Affiliations:** 1 Department of Molecular Neurology, Uniklinikum Erlangen, Friedrich-Alexander-Universität Erlangen-Nürnberg, Erlangen, Germany; 2 Department of Neurology, Medical University of Innsbruck, Innsbruck, Austria; 3 Department Artificial Intelligence in Biomedical Engineering, Friedrich-Alexander-Universität Erlangen-Nürnberg, Erlangen, Germany; 4 Digital Medicine, Luxembourg Center for System Biomedicine (LCSB), University of Luxembourg, Esch-sur-Alzette, Luxembourg; 5 École Polytechnique Fédérale de Lausanne (EPFL), Lausanne, Switzerland; 6 Service of Neurology, Department of Clinical Neurosciences, Lausanne University Hospital and University of Lausanne, Lausanne, Switzerland; 7 Center for Parkinson and Movement Disorders, Neurology, Reha Rheinfelden, Rheinfelden, Switzerland; 8 Department of Neurology, Provincial Hospital of Bolzano, Bolzano, Italy; 9 Paracelsus Private Medical University, Salzburg, Austria; 10 Fraunhofer Institute for Integrated Circuits IIS, Erlangen, Germany

**Keywords:** Gait analysis, Atypical parkinsonism, Parkinson’s disease, Wearable sensors, Mobility assessment

## Abstract

**Background and aim:**

Parkinsonian disorders are hallmarked by gait and balance impairments. Atypical parkinsonian disorders (APD) develop postural instability with falls and gait disorders early on. Sensor-based gait recordings provide objective data in hospital and everyday life, improving mobility assessment accuracy. However, the impact of duration and distance of instrumented assessments on construct validity remains unclear. This exploratory study aims to evaluate the construct validity of gait assessments compared with clinical, functional, and patient-reported scores.

**Methods:**

The multi-centered Mobility_APP study recruited 43 PD and 49 APD patients. Among others, the Berg Balance Scale (BBS) and the postural instability and gait difficulty score (PIGD) were collected. Sensor-based gait parameters were captured during standardized 2 × 10 m and 2-min walk tests (2MWT) in the hospital and for 1 day of physical activity monitoring (PAM) at home. PAM was categorized by short (10–30 s), medium (30–60 s), and long (≥ 60 s) walking bouts (WB). Spearman correlations were applied to investigate associations between scores.

**Results:**

Mean gait velocity (GV) and stride length correlated more strongly with functional, clinical, and patient-reported scores in 2MWT than in 2 × 10 m. Additionally, the GV variability in the 2MWT correlated with BBS and PIGD (*r* = │0.3–0.7│), but was less prominent in 2 × 10 m (*r* = │0.0–0.5│). In PAM, GV of long WB correlated more strongly with the PIGD (*r* = │0.5–0.6│) than short WB (*r* = │0.2–0.4│).

**Conclusion:**

The 2MWT tended to show the highest construct validity. PAM offered complementary but weaker correlations, highlighting that PAM provides novel insights into daily life mobility of APD patients.

**Supplementary Information:**

The online version contains supplementary material available at 10.1007/s00415-026-13652-0.

## Introduction

Parkinson’s disease (PD) is a neurodegenerative disorder affecting motor and non-motor functions impacting a broad range of activities of daily living [1]. Similar impairments are shown in atypical parkinsonian disorders (APD) such as multiple system atrophy of Parkinson’s type (MSA-P) and progressive supranuclear gaze palsy-type Richardson syndrome (PSP-RS), where they are even more pronounced due to a poor levodopa response and a faster disease progression [1–3].

In addition to parkinsonian symptoms such as rigidity, bradykinesia, and gait impairments, MSA-P is hallmarked by autonomic failure, impairments in health-related quality of life (QoL), and a tendency to fall [1, 2]. PSP-RS is mainly characterized by vertical supranuclear gaze palsy, cognitive impairments, and severe postural instability leading to falls [3].

Previous studies described different gait patterns for PD, MSA-P, and PSP-RS. [4–8] PD patients show a narrow-based gait without instability in early disease stages [5]. Shuffling, step length, and regularity of gait are slightly reduced in the early stages compared to healthy controls, but become more pronounced with disease progression [9]. In contrast, MSA-P and PSP-RS patients have a broad-based, irregular gait reflected in higher coefficients of variation (CV) for stride, swing and stance time, gait velocity (GV), and stride length (SL) [4, 5, 7]. Although the gait pattern of PSP-RS is very unstable, it has been described as “reckless walking” or “drunken sailor” with rapid movements and abrupt stops and turns [5]. Apart from these findings, it is more difficult to discriminate MSA-P and PSP-RS patients based solely on gait parameters [6]. These patterns were identified using sensor-based gait systems during standardized tests in a clinical setting [4, 6, 7, 9, 10]. However, little is known about mobility in daily living for PD [11] and especially for MSA-P and PSP-RS.

The Mobility_APP study [12] evaluated gait patterns, both in standardized hospital settings and under real-world conditions using objective measurements in a large cohort of patients with Parkinson's disease and APD. The study's conclusions raised new questions about the clinical validity of these different testing scenarios—particularly with regard to comparing results from controlled clinical settings with measurements taken under real-world conditions—and underscored the need to further investigate their potential for developing innovative treatment approaches and outcome measures. During standardized tests, patients performed short bouts of walking (2 × 10 m) and longer bouts during a 2-min walk test (2MWT) to evaluate gait capacity. Daily-life gait recordings capture short, medium, and long walking bouts (WB), providing insights into both walking duration and context [13]. Short WB are more common indoors, while long WB often occur outdoors. However, it is not well understood, which duration is clinically the most meaningful. Therefore, this manuscript aims to assess the construct validity for pre-selected instrumented gait assessments through clinical, functional, and patient-reported scores of gait impairment and postural instability in an exploratory approach, resulting in data-driven recommendations for assessing gait in PD and APD.

## Methods

### Participants

The multi-centered study group recruited patients via the Movement Disorder Outpatient Units in Innsbruck, Austria; Erlangen, Germany; and Bolzano, Italy as part of the Mobility_APP trial (more details under NCT04608604)[12]. The study was approved by the local ethics committees (IRB numbers: 26_20 B (Erlangen), 1290/2020 (Innsbruck), 49-2015 (Bolzano)), and all patients had to provide written informed consent. The research question mentioned above was predefined as an exploratory substudy as part of the Mobility_APP project. Inclusion criteria for the Mobility_APP study required patients to be 30–80 years old with a diagnosis of possible or probable MSA-P, possible or probable PSP-RS or PD according to current clinical diagnostic criteria [14–16]. Their antiparkinsonian and anti-OH (orthostatic hypotension) medication had to remain stable for at least 4 weeks prior to enrollment. Patients with medical conditions significantly affecting parkinsonian symptoms were excluded. Individuals with Hoehn and Yahr stage higher than 3, or individuals with secondary causes of autonomic failure or parkinsonism (e.g., diabetic autonomic neuropathy, bladder surgery), were excluded. Furthermore, patients diagnosed with dementia under DSM-V criteria or those who had participated in another clinical trial affecting the study outcomes were excluded. During the hospital visit, assessments were conducted in the patients’ medical ON state in those receiving levodopa therapy.

### Clinical data

#### Clinical scores

Certified assessors rated disease severity according to the Movement Disorder Society-Unified Parkinson's Disease Rating Scale (MDS-UPDRS) [17]. The patient-reported Part II of the MDS-UPDRS captures motor experiences in daily living, while Part III evaluates motor function, including the postural instability and gait difficulty (PIGD) score [18], to assess mobility-related impairments rated by a clinician. Furthermore, cognitive function was evaluated using the Montreal Cognitive Assessment (MoCA)[19] to screen for cognitive deficits commonly associated with PD and APD.

#### Functional scores

Impaired balance and mobility and a high risk of falls are key factors in APD and PD, significantly affecting gait. In this trial, balance was assessed using the Berg Balance Scale (BBS)[20] and functional mobility was tested with the timed up and go (TUG) test over a walking distance of 3 m. [21]

#### Patient-reported scores

To incorporate the patients’ perspective, quality of life (QoL) and physical activity were assessed through patient inquiries. The impact of motor and non-motor symptoms on QoL was evaluated using the Parkinson’s Disease Questionnaire (PDQ-8) including physical, emotional, and social aspects [22]. The long form of the International Physical Activity Questionnaire (IPAQ) measured physical activity levels, including frequency, duration, and intensity with a recall period of 7 days [23] to gain insights of the patients’ physical activity behavior in daily living.

An overview of all scores can be found in the supplements table S1.

### The mobile GaitLab sensor system

**Fig. 1:**
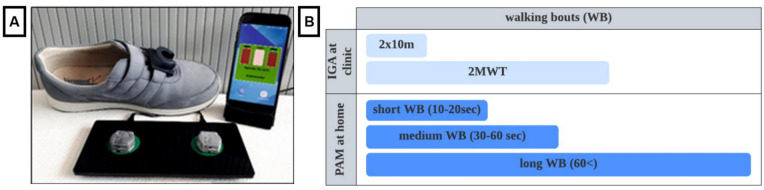
Mobile GaitLab system (**A**). Walking bouts (WB) recorded at the clinic and at home (**B**)

The mobile GaitLab system provided by Portabiles HealthCare Technologies GmbH (Erlangen, Germany) was used for instrumented gait analysis (IGA) at clinic and physical activity monitoring (PAM) at home. The system includes two inertial measurement units (IMU), each containing a 3D accelerometer and 3D gyroscope recording at a sampling rate of 102.4 Hz. The IMUs are attached to the patient’s instep of their shoes during IGA at clinic and PAM at home as shown in Fig. 1A. The sensor-based gait analysis system was technically and clinically validated as described. [7, 24, 25]

### Instrumented gait analysis at clinic

Patients performed a 2 × 10 m walking test (2 × 10 m) (Fig. 1B). This test required one turn, while walking at preferred speed without stops. The 2 × 10 m walking distance was chosen based on experiences from previous gait analysis projects [7] and to limit the burden for severely affected patients, Additionally, the patients completed a 2 min walk test (2MWT) along a 20- to 25-m path, turning at each end and aiming to achieve the maximum distance [26]. Four patients (MSA-P: *n* = 1, PSP-RS: *n* = 3) completed the 2MWT on a 10-m path due to logistical constraints on the day of the recording. Statistical analyses were conducted both including and excluding these patients, revealing no significant differences in the results. Consequently, these patients were retained in the final analysis. A pipeline based on the Gaitmap package [27] calculated more than ten parameters including the CV for each parameter. Furthermore, turning strides were automatically excluded by the algorithm based on the turning angle greater than 25 degrees and stride length shorter than 0.3 m [28]. As the algorithm was not able to detect distinct turning strides of the APD patients (see supplementary material figure S2), the turnings were excluded for all patients for maintaining consistency and better comparability. Included were solely straight steps defined as constant steps, and acceleration and deceleration defined as non-constant steps.

### Physical activity monitoring at home

Patients wore identical shoe sensors at home for 7 days prior to the hospital visit. They initiated the recording each morning upon awakening and stopped it in the evening when going to sleep; during the night the devices needed to be charged. The objective was to record their daily activities at least 8 h per day. For this analysis, the day closest to the hospital visit with at least 8 h of wearing time was selected to ensure the most accurate comparison with the clinic-based gait analysis. GV(CV) and SL(CV) were extracted from sensor signals using a validated pipeline [29]. The WB performed in daily life were divided into short, medium, and long sequences of consecutive steps (Fig. 1B). Short WB were defined by a duration of 10–30 s, medium WB lasted 30–60 s, and long WB included all WB longer than 60 s. [13] The detailed study protocol including a description of the extraction of all gait parameters has been published previously [12].

### Statistical analysis

Statistical analyses were conducted using IBM SPSS Statistics version 28 (IBM Corp., Released 2021. IBM® SPSS® Statistics for Windows, Version 28.0.0.0, Armonk, NY, USA: IBM Corp.). The Shapiro–Wilk test assessed the normality of all parameters, and Levene’s test examined homogeneity of variance. Since several parameters did not follow a normal distribution, a conservative approach utilizing non-parametric methods was applied. Spearman's correlation coefficient evaluated relationships between gait parameters recorded during different WB at home and in the clinic, and the clinical, functional, and patient-reported outcomes within each patient group. R-values below 0.3 were considered as low, r-values between 0.3 and 0.5 as moderate, and over 0.5 as strong [30]. A p-value below 0.05 indicated significance. The calculated p-value was adjusted with the Benjamini–Hochberg correction to control the false discovery rate in multiple testing. For IGA, a bootstrap procedure was performed to assess the stability of the correlations due to the relatively small subgroup sizes. Bias-corrected percentile bootstrap confidence intervals (95% CI) were computed using 5,000 resamples for the 2MWT and the 2 × 10 m test. To statistically test whether the 2MWT provided stronger correlations than the 2 × 10 m test, a paired bootstrap approach was used: in each resample, Spearman’s r values for both gait tests and their difference (Δr = r_{2MWT} − r_{2 × 10 m}) were calculated. The resulting bootstrap distribution of Δρ was used to derive a 95% confidence interval (CI) and a one-sided *p*-value. The superiority of 2MWT over 2 × 10 m was considered statistically supported if the CI was not 0 and the *p*-value was < 0.05. Otherwise, results were described as a tendency toward stronger correlations for 2MWT. Bootstrap analysis was performed in Python (version 3.11.7).

To examine differences in the number of WBs across the respective WB lengths of PAM, as well as differences in the number of steps performed within short, medium, and long WB, the Kruskal–Wallis test was applied. To further explore differences between gait assessments, a stride-by-stride analysis of GV was conducted. Therefore, the range of GV, defined as the difference between the slowest and fastest strides within each gait assessment, was calculated for each patient. Additionally, the GV for each stride was graphically presented for the 2 × 10 m and 2MWT in one patient per disease entity to illustrate how the representation of gait capacity varies depending on the specific gait test. Gait capacity represents the maximum level of walking performance a person could achieve under ideal conditions, as opposed to their actual walking ability in daily life [31].

To account for potential influences of cognitive deficits, a partial correlation was performed for the PSP-RS cohort controlled for cognition as a confounder (MoCA). Since no cognitive deficits were present in MSA-P and PD patients based on the MoCA score, it was not expected that the correlations between clinical, functional, and patient-reported scores and gait parameters were affected by the confounder analysis.

## Results

In total, 106 patients were recruited. For this subanalysis, the data of 14 patients were excluded due to not meeting the criteria of sufficient wearing time of the sensors at home, at least 1 day including 8 h. In total, the data of 43 PD, 28 MSA-P, and 21 PSP-RS patients were analyzed. Patient characteristics are presented in Table 1 and gait characteristics in Table 2.

**Table 1:** Patient characteristics for each disease entity

	PD (*n* = 43)	MSA-P (*n* = 28)	PSP-RS (*n* = 21)	*p*-value	Effect Size	*p*-value	*p*-value	*p*-value
	Mean (SD)	Mean (SD)	Mean (SD)		(Ꞃ^2^)	PD – MSA-P	PD—PSP-RS	MSA-P—PSP-RS
Age, years	68.4 (8.9)	64.6 (7.4)	69.5 (6.7)	0.056	0.04	NA	NA	NA
Gender, m/f	23 / 20	14/14	14/7	0.241	0.01	NA	NA	NA
BMI, kg/m^2^	26.3 (4.1)	24.0 (4.0)	26.2 (2.9)	**0.017**	**0.08**	*	**–**	*
Disease duration, years	7.9 (5.1)	4.6 (2.3)	3.4 (1.8)	** < 0.001**	**0.16**	***	***	**–**
Hoehn and Yahr	2.0 (0.5)	2.6 (0.5)	2.7 (0.6)	** < 0.001**	**0.37**	***	***	**–**
LEDD, mg/d	546 (401)	479 (409)	541 (346)	0.479	0	NA	NA	NA
Faller, %	7	39.3	61.9	** < 0.001**	**0.15**	*	***	**–**
MDS-UPDRS-III	21.3 (10.8)	40.0 (13.1)	33.6 (11.9)	** < 0.001**	**0.35**	***	***	**–**
UMSARS	NA	36.86 (11.40)	NA	NA	NA	NA	NA	NA
PSP-RS	NA	NA	33.45 (13.32)	NA	NA	NA	NA	NA
MoCA	26.3 (3.0)	25 (4.1)	22.9 (3.4)	** < 0.001**	**0.14**	**–**	***	*

*SD* standard deviation; *LEDD* levodopa equivalent daily dose; *MDS-UPDRS-III* Motor Score of the MDS-Unified Parkinson’s disease Rating Scale; *UMSARS* Unified Multiple System Atrophy Rating Scale; *PSP-RS* Progressive Supranuclear Palsy Rating Scale; *MoCA* Montreal Cognitive Assessment, Kruskal–Wallis test: *p < 0.05; **p < 0.01; ***p < 0.001; NA not applicable (adjusted for false discovery rate (FDR) according to Benjamini–Hochberg’s correction.)

Bold numbers represent significant differences (p<0.05)

**Table 2:** Gait parameters for each disease entity

	PD (n = 43)	MSA-P (n = 28)	PSP-RS (n = 21)	*p*-value	Effect Size	*p*-value	*p*-value	*p*-value
	Mean (SD)	Mean (SD)	Mean (SD)		(*Ꞃ*^2^)	PD – MSA-P	PD—PSP-RS	MSA-P—PSP-RS
IGA at clinic 2 × 10 m								
GV, m/s	1.19 (0.18)	0.90 (0.21)	0.88 (0.25)	** < 0.001**	**0.34**	***	***	**–**
SL, m	1.29 (0.18)	1.03 (0.22)	1.06 (0.23)	** < 0.001**	**0.26**	***	***	**–**
GV, CV	0.09 (0.05)	0.11 (0.05)	0.11 (0.05)	**0,016**	**0.07**	*	*	**–**
SL, CV	0.06 (0.03)	0.09 (0.05)	0.09 (0.05)	**0.002**	**0.13**	***	*	**–**
IGA at clinic 2MWT								
GV, m/s	1.27 (0.30)	0.89 (0.27)	0.89 (0.27)	** < 0.001**	**0.29**	***	***	**–**
SL, m	1.33 (0.22)	1.01 (0.27)	1.06 (0.26)	** < 0.001**	**0.27**	***	***	**–**
GV, CV	0.07 (0.03)	0.10 (0.04)	0.12 (0.06)	** < 0.001**	**0.30**	***	***	**–**
SL, CV	0.05 (0.02)	0.09 (0.06)	0.09 (0.07)	** < 0.001**	**0.21**	***	***	**–**
PAM at home short WB								
GV, m/s	0.51 (0.16)	0.45 (0.16)	0.43 (0.12)	0.110	0.05	NA	NA	NA
SL, m	0.69 (0.20)	0.60 (0.20)	0.62 (0.18)	0.110	0.04	NA	NA	NA
GV, CV	0.38 (0.09)	0.35 (0.10)	0.38 (0.08)	0.159	0.03	NA	NA	NA
SL, CV	0.30 (0.09)	0.30 (0.12)	0.30 (0.07)	0.519	0.00	NA	NA	NA
PAM at home medium WB								
GV, m/s	0.65 (0.23)	0.53 (0.23)	0.57 (0.20)	0.156	0.03	NA	NA	NA
SL, m	0.85 (0.24)	0.70 (0.25)	0.76 (0.23)	0.156	0.05	NA	NA	NA
GV, CV	0.34 (0.11)	0.37 (0.09)	0.33 (0.08)	0.170	0.02	NA	NA	NA
SL, CV	0.30 (0.13)	0.35 (0.13)	0.29 (0.08)	0.170	0.02	NA	NA	NA
PAM at home long WB								
GV, m/s	0.96 (0.28)	0.74 (0.26)	0.84 (0.24)	**0.026**	**0.09**	**	**–**	**–**
SL, m	1.10 (0.24)	0.87 (0.31)	1.01 (0.23)	**0.026**	**0.08**	**	**–**	**–**
GV, CV	0.22 (0.13)	0.27 (0.12)	0.22 (0.09)	0.151	0.02	NA	NA	NA
SL, CV	0.19 (0.13)	0.24 (0.14)	0.17 (0.08)	0.151	0.02	NA	NA	NA

*SD* standard deviation; *GV* gait velocity; *SL* stride length; *CV* coefficient of variability; *IGA* instrumented gait assessment; *PAM* physical activity monitoring; Kruskal–Wallis test: *p < 0.05; **p < 0.01; ***p < 0.001; NA not applicable (adjusted for the false discovery rate (FDR) according to Benjamini–Hochberg’s correction)

Bold numbers represent significant differences (p<0.05)

The analysis in this manuscript focused solely on GV and SL and their CVs, as these parameters were shown in a previous paper of our group to be the most relevant for APD [7]. Further, GV and SL (+ CV) showed the most relevant patterns across different WB in the initial exploratory analyses. In contrast, other gait parameters such as swing time, stance time, stride time, and their CVs did not demonstrate meaningful correlation patterns with most clinical, functional, or self-reported scores. Overall, only two significant correlations emerged within the 2MWT: for MSA-P, swing time (CV) correlated with the BBS, and for PSP-RS, swing time (CV) correlated with the PIGD. No significant correlations were found for PD (see table S4).

GV and SL and their CVs show significant differences for the 2 × 10 m and 2MWT between PD and MSA-P, and PD and PSP-RS. PD patients walk significantly faster with longer strides and greater stability compared to APD patients. For the PAM analysis, the only difference was found between PD and MSA-P within GV and SL of long WB. Overall, no significant difference was found between MSA-P and PSP-RS.

### Assessing construct validity of instrumented gait assessments at the clinic

Comparing gait parameters in MSA-P from the 2MWT and the 2 × 10 m with clinical, functional, and self-reported scores, moderate to strong correlations were observed for IGAs (*r* = │0.3–0.8│, see Table 3). Interestingly, the strongest correlations were found for both IGAs between GV (*r* = 0.8, *p* < 0.05) and SL (*r* = 0.8, *p* < 0.05) with the BBS. However, the correlations between the CVs and all scores were tendentially stronger in the 2MWT (*r* = │0.3–0.7│) compared to the 2 × 10 m (*r* = │0.1–0.6│), with strongest correlations between SL(CV) and BBS (*r* = 0.7, *p* < 0.05).

**Table 3:**
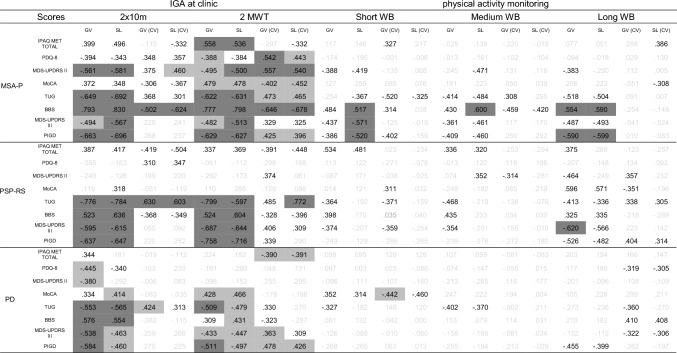
Spearman correlation between clinical, functional, and patient-reported scores and gait parameters (mean and CV) of the different WB lengths

*IGA* instrumented gait analysis; *GV* gait velocity; *SL* stride length; *CV* coefficient of variation; *IPAQ* International Physical Activity Questionnaire; *PDQ-8* Parkinson’s Disease Questionnaire; *MDS-UPDRS* Movement Disorder Society Unified Parkinson’s Disease Rating Scale; *MoCA* Montreal Cognitive Assessment; *TUG* timed up and go test; *BBS*: Berg Balance Scale; *PIGD*: postural instability and gait difficulty

*P*-value adjusted according to Benjamini–Hochberg correction

Legend: ; *p* > 0.05 and *r* > .300; ;

Moderate to strong correlations were observed for PSP-RS between GV and SL with all clinical and functional scores, with the exception of the MoCA. Markedly, correlations were similar for the 2 × 10 m (*r* = │0.5–0.8│) and the 2MWT (*r* = │0.5–0.8│). Strongest correlations were observed between GV and SL with TUG and PIGD (*r* = 0.8, *p* < 0.05) for both IGAs. However, higher correlations were observed between SL(CV) and GV(CV) of the 2MWT and all functional and clinical scores (*r* = │0.3–0.8│) compared to 2 × 10 m (*r* = │0.1–0.6│), with strongest correlations between SL(CV) and TUG (*r* = 0.8, *p* < 0.05). No significant correlations were found between any patient-reported scores and the gait parameters.

Compared to MSA-P and PSP-RS, the correlations between the gait parameters and the clinical, functional, and self-reported scores were weaker in PD. GV and SL extracted from the 2 × 10 m showed moderate correlations with all clinical and functional scores (*r* = │0.3–0.6│, see Table 3), which were similar compared to 2MWT (*r* = │0.3–0.5│), with the strongest correlations between GV and PIGD (2 × 10 m: *r* = 0.6, *p* < 0.05; 2MWT: *r* = 0.5, *p* < 0.05) for both IGAs. In contrast to the 2 × 10 m test (*r* = │0.0–0.4│), the CV for GV and SL in the 2MWT moderately correlated with clinical and functional scores (*r* = │0.2–0.5│). The strongest correlation for the 2 × 10 m was observed between GV (CV) and TUG (*r* = 0.4, *p* < 0.05), whereas the strongest correlation for the 2MWT was shown between GV (CV) and PIGD (*r* = 0.5, *p* < 0.05).

Furthermore, the paired bootstrap comparisons showed that correlations of gait parameters from the 2MWT tended to be stronger than those of the 2 × 10 m across patient subgroups. However, none of these differences reached statistical significance. Thus, while the 2MWT tended to yield stronger correlations with clinical and patient-reported outcomes, superiority over the 2 × 10 m could not be statistically confirmed in any subgroup.

### Assessing construct validity of different walking bout lengths of PAM at home

**Fig. 2:**
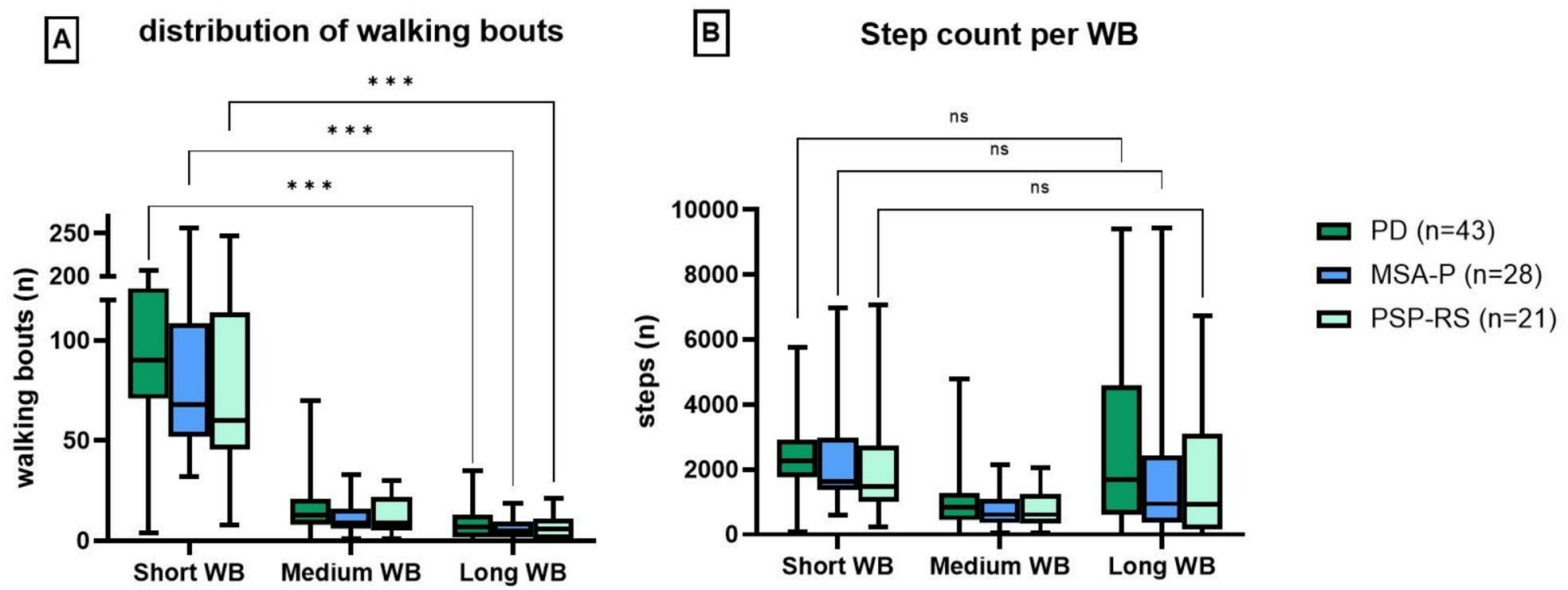
Number of recorded walking bouts at home (**A**). Total number of steps recorded within the bouts (**B**). Short WB = 10–30 s, medium WB = 30–60 s, long WB > 60 s. *p < 0.05; **p < 0.01; ***p < 0.001; *ns* not significant

Patients across all diagnoses performed significantly fewer total steps during medium WB (MED = 674 steps) compared to short WB (MED = 1980 steps) and long WB (MED = 1388 steps) in daily life (see Fig. 2B). Most steps occurred during short WB, with no significant difference to long WB across diagnoses. Interestingly, four MSA-P, three PSP-RS, and four PD patients did not perform any long WB. Since less steps were recorded for medium WB, the comparison between similar databases of long and short WB is more reliable.

In MSA-P, GV and SL demonstrated moderate correlations with all functional and clinical scores across short and long WB (*r* = │0.3–0.6│), with the exception of MoCA. The strongest correlations were observed between GV and SL with PIGD for long WB (*r* = 0.6, *p* < 0.05). GV(CV) and SL(CV) did not show any specific correlations with PIGD. In contrast to MSA-P, PSP-RS and PD showed larger differences between short and long WB in home recordings. For short WB, weak to moderate correlations were observed (*r* = │0.0–0.4│) across all functional and clinical scores, whereas a tendency toward stronger correlations between GV and SL of long WB with these scores (*r* = │0.1–0.6│) were observed. The strongest correlation was observed between GV and MDS-UPDRS-III (*r* = 0.6, *p* < 0.05) in PSP-RS. GV(CV) and SL(CV) for long WB showed moderate correlations with PIGD and TUG in PSP-RS. In PD, GV(CV) and SL(CV) showed stronger correlations with PDQ-8, MDS-UPDRS-III, and BBS as the WB duration increased (short WB: *r* = │0.0–0.2│; long WB: *r* = │0.3–0.4│).

Comparing correlations between recordings at clinic and in daily life across all disease entities, IGA at clinic showed stronger associations between GV and SL with clinical/functional scores for both gait tests than recordings from daily living, regardless of WB length. Patient-reported outcome measures showed only a few moderate correlations with GV and SL recorded in the clinic. In comparison, correlations between patient-reported outcome measures and GV and SL in daily life were even weaker.

### Influence of cognitive deficits on correlations

After controlling for MoCA in the PSP-RS cohort, the correlations between gait parameters (both IGA and PAM) and MDS-UPDRS-II and III, BBS, and PIGD improved to moderate to strong (supplementary material table S3). Notably, the correlations between GV(CV) of the 2MWT and MDS-UPDRS-II, as well as between gait parameters and the clinical and functional scores, became stronger when adjusted for MoCA. Overall, nearly all correlations between 2MWT gait parameters and the scores reached moderate to strong levels.

### Stride-by-stride analysis at the clinic and in daily living

**Fig. 3:**
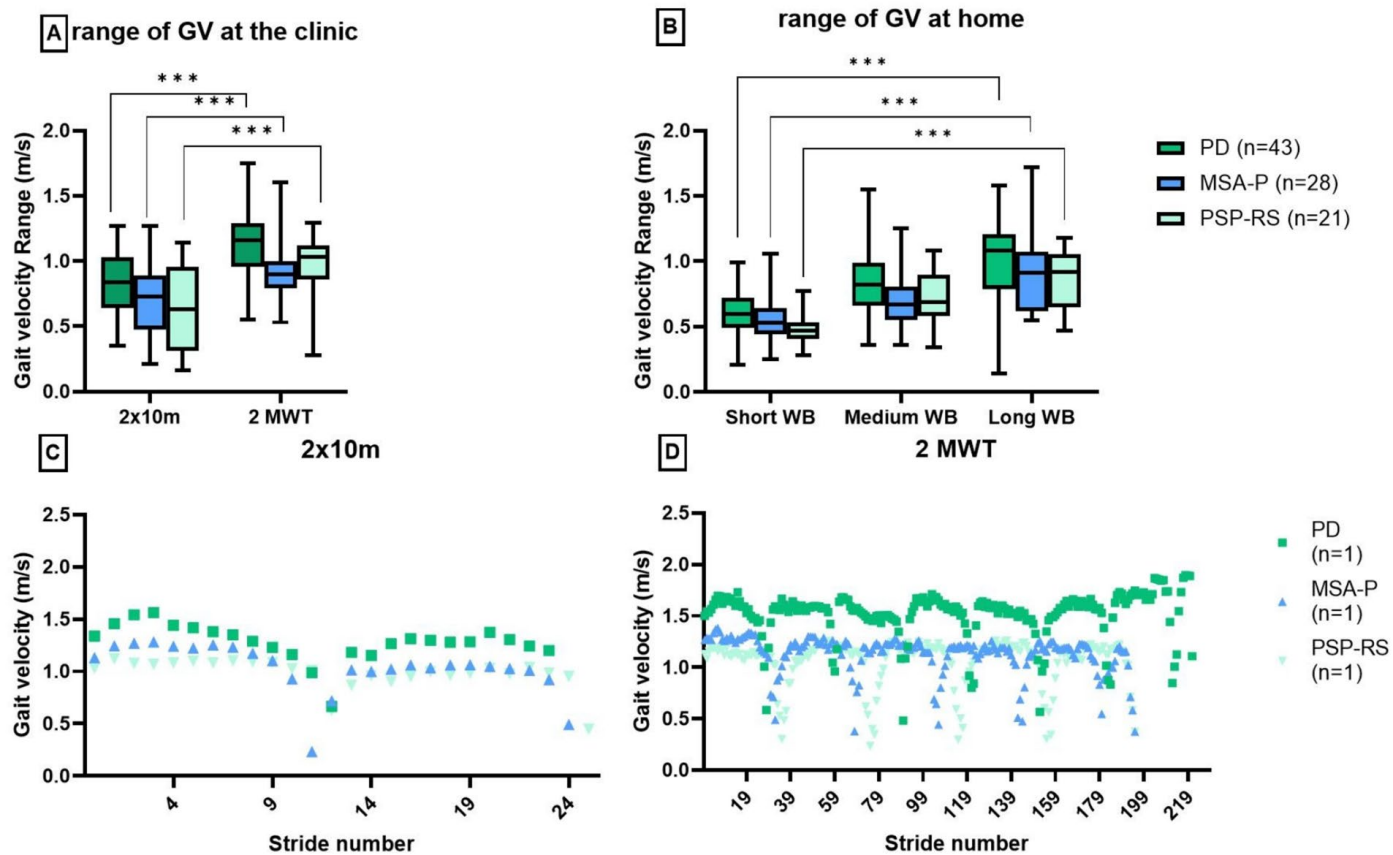
Range between the slowest and fastest steps performed during IGA (**A**) and PAM at home (**B**). Single stride analysis of gait velocity during 2 × 10 m (**C**) and 2MWT (**D**) at clinic. Each disease subgroup is represented by one patient as an example (B and C). *p < 0.05; **p < 0.01; ***p < 0.001

To better understand the reported correlations, GV of the single strides within IGA at clinic and PAM at home were analyzed for all cohorts and in single cases of each disease subgroup. This analysis revealed a significantly higher range of the GV (Fig. 3A) within the 2MWT for APD and PD compared to the 2 × 10 m. Compared to 2 × 10 m, 2MWT included more constant and non-constant steps across all diseases, reflecting different test conditions. Due to a higher number of turnings, more acceleration and deceleration steps were recorded. While for the 2 × 10 m, the GV of the single steps seemed to be similar for MSA, PSP-RS, and PD, a more distinct pattern was observed in the 2MWT, showing differences between GV patterns of APD and PD (Fig. 3D).

These findings were reflected by the gait in PAM at home. A significantly higher range of GV was found for long WB compared to short WB for each subgroup: MSA-P (long WB MED = 0.91 m/s; short WB MED = 0.58 m/s), PSP-RS (long WB MED = 0.87 m/s; short WB MED = 0.48 m/s), and PD (long WB MED = 1.02 m/s; short WB MED = 0.63 m/s) (Fig. 3B). Stride-by-stride analysis of 2MWT and long WB revealed greater GV variety, and more recorded steps, making them more suitable for assessing overall gait capacity (2MWT) and gait performance (long WB). This supports the stronger correlations between the CVs and especially PIGD for the 2MWT compared to 2 × 10 m for all disease entities. Similarly, GVs of long WB appeared to be more relevant in association with PIGD and BBS compared to short WB in PD and PSP-RS.

### Differences in gait velocity according to H&Y stages

**Fig. 4:**
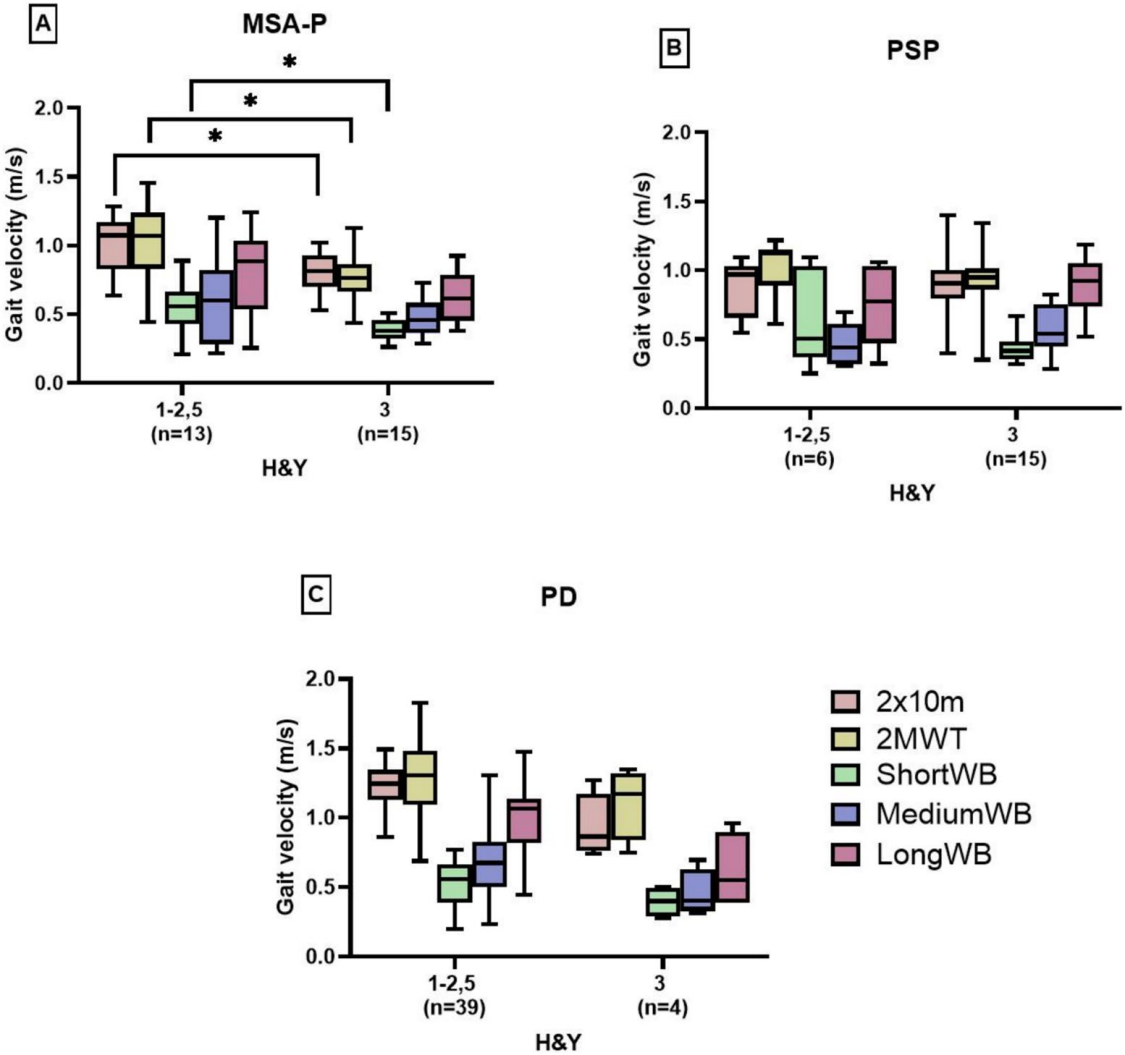
Gait velocity per WB compared between patients with (H&Y 3) and without (H&Y 1–2,5) impaired balance and postural instability. *p < 0.05; **p < 0.01; ***p < 0.001

Since gait is highly affected by postural instability, comparing H&Y stages (H&Y 1–2.5 without and H&Y 3 with postural instability) provides additional insights into gait performance across gait assessments. A significant decrease of GV was observed for MSA-P patients when comparing H&Y stage 1–2.5 and H&Y stage 3 for 2 × 10 m (-21%), 2MWT ( – 23%), and short WB at home ( – 26%) (Fig. 4A). However, no significant differences were observed between H&Y stages for medium and long WB. Interestingly, PSP-RS patients showed no decrease, as GV remained similar across H&Y groups. GV changes in PD patients were similar, but comparability was limited by the small H&Y 3 sample size (*n* = 4 vs. *n* = 39 in H&Y 1–2.5) in PD and the small H&Y1-2,5 sample size (*n* = 6 vs. *n* = 15 in H&Y3) in PSP-RS.

## Discussion

This exploratory study aimed to assess the construct validity of pre-selected instrumented gait assessments and to identify the gait test that is best associated with clinical, functional, and patient-reported scores of gait impairment and postural instability in PD and APD, resulting in potential recommendations for assessing gait in the future. Overall, the analysis revealed stronger correlations between longer walking bouts and functional, clinical, and patient-reported scores compared to shorter WB.

### Construct validity of gait assessments in a clinical setting: highlighting the benefits of the 2MWT

This study showed a tendency toward a higher construct validity of the 2MWT compared to the 2 × 10 m in assessing gait patterns and gait capacity in individuals with APD and PD. Therefore, the 2MWT may be a preferable option for evaluating gait patterns in this population. However, for more severely impaired patients unable to walk for 2 min due to physical limitations, the 2 × 10 m remains a meaningful alternative, providing valuable data, as shown in our previous work [7]. Both standardized gait tests seem to contain gait information that is not reflected in the clinical, functional, and patient-reported scores. These objective gait features need further investigation.

Regarding the number of collected steps, CVs derived from the 2 × 10 m should be interpreted cautiously. As reported, reliable CV calculation is more accurate if at least 30 steps were recorded [10]. Most patients performed fewer than 30 steps during the 2 × 10 m, potentially compromising CV-based analyses. As CV is considered to predict fall risk [32, 33], which is crucial for APD, this underlines the advantage of performing the 2MWT whenever feasible.

Moreover, recent studies have identified irregular walking patterns predictive for falls in PSP-RS [34]. Falls mostly occur during gait initiation, stopping, or turning, when postural stability is challenged rather than during steady-state walking [35]. Since the 2MWT captures more non-constant steps, this assessment appears more relevant than the 2 × 10 m, which collects significantly less steps. This aligns with findings of Nguyen et al. (2019), suggesting that a more detailed and separate analysis of constant and non-constant steps, but also turnings, provides deeper insights into gait impairments in PD [36]. This is further supported by stronger correlations between gait parameters and functional scores in the 2MWT compared to the 2 × 10 m (e.g., BBS/GV, 2MWT: *r* = 0.3–0.7; 2 × 10 m: *r* = 0.1–0.5). As turning steps were not subject of this analysis, it is important to consider turnings in future studies to investigate their relevance in depth as indicated by recent findings in the literature [37].

Overall, PD patients exhibited milder motor impairments and weaker correlations with clinical and functional scores compared to APD. Previous studies have shown that PD patients in early disease stages present many results of gait parameters similar to healthy controls, with weaker correlations to clinical ratings [9, 38]. As PD patients often present bradykinesia and tremor first, postural instability is not fully reflected in the clinical scores. The pull test of the MDS-UPDRS, to measure postural instability, is highly rater dependent and therefore needs to be supported by more objective measurements [39]. These findings emphasize the need to differentiate gait patterns across disease stages to better understand changes in gait parameters and postural instability over time. This knowledge may support clinicians in disease diagnosis and in predicting disease progression early on.

By analyzing GV in the 2MWT grouped by H&Y stage, a shift of gait patterns between patients with and without postural instability was observed. Interestingly, postural stable PSP-RS and MSA-P patients had a comparable GV, whereas postural stable PD patients walked significantly faster. However, among postural instable patients, PSP-RS patients walked as fast as PD patients, while MSA-P patients were significantly slower. This shift supports the characterization of PSP-RS gait patterns as “reckless” or “drunken sailor” [5], as their GV remained higher even with postural instability. However, the PSP-RS cohort of H&Y1-2,5 (*n* = 6) was significantly smaller than the cohort of H&Y3 (*n* = 15); therefore, the results should be interpreted with caution and need further investigation. Additionally, cognitive deficits should be further investigated, as analysis revealed that the MoCA functioned as a suppressor for correlations between clinical, functional, and patient-reported scores and gait parameters. Dual tasking can give more insights into actual interactions between scores and gait patterns as well as a deeper understanding of the pathology [40].

### Construct validity of gait assessments under real-life conditions: novel insights for clinicians

For the first time, an in-depth recording of real-life physical activity behavior was conducted in a large APD cohort, providing valuable insights into mobility in daily life. For all patients, gait parameters derived from longer WB during PAM in daily life showed stronger correlations with clinical, functional, and patient-reported scores compared to short WB, indicating greater construct validity.

As indicated in the literature for PD [41], moderate to strong correlations were observed in all patient groups, particularly between SL and GV with clinically established measures (BBS, MDS-UPDRS-III, PIGD). However, compared to standard conditions in the hospital, environmental influences (e.g., weather, social interactions, walking surfaces) and differences in physical activity behavior should be considered when interpreting real-life gait patterns. This aligns with previous findings suggesting that the environment impacts patients’ physical activity, though specific environmental factors affecting gait remain unclear [11]. Importantly, PAM at home offers a unique opportunity to capture personalized behavior influenced by disease-specific motor impairments and real-world conditions. PAM at home goes beyond reflecting results from established scores, but provides further insights into daily mobility of the patient by measuring real walking performance [11] which cannot be recorded during IGA at the clinic where only gait capacity can be measured with the 2MWT [26]. To correctly interpret these insights into patients’ real lives, future studies must develop a comprehensive understanding of environmental effects [41] and the recently developed concept of patient’s walking experience [42]. The latter involves social, emotional, mental, and physical aspects, which future research should consider.

### Limitations

The main limitation to this analysis was the exclusion of turning strides. The algorithm that was used for this study can detect turnings in PD; however, it is not trained to detect turnings in most APD patients and the exclusion of turnings highlighted the need to develop an algorithm specifically for APD. The dataset of the Mobility_APP study delivers important insights for a future development of such an algorithm.

As MSA-P and PSP-RS progress rapidly, matched PD patients exhibited lower impairments according to the H&Y, the MDS-UPDRS motor score, and the BBS in which we recognized a ceiling effect for the PD cohort, limiting the scope of group comparisons. The ceiling effect of the BBS in PD patients may have reduced score variability, which could have an influence on the lower correlations with gait parameters that were observed for the PD cohort. Notably, four PD patients were categorized as having postural instability (H&Y3), making comparisons between groups with different sample sizes prone to bias and potentially limiting conclusions.

Additionally, monitoring physical activity for 1 day, with a minimum of 8 h, may not fully capture daily gait patterns. Correlating PAM during 1 day with patient-reported physical activity (IPAQ) over 1 week should be interpreted with caution due to the different time span. Collecting WB data over multiple days could provide a more comprehensive understanding of patients’ physical activity, potentially strengthening these correlations. Moreover, comparisons between hospital-based supervised gait tests and home-based recordings remain limited, as environmental factors influence physical activity behavior in ways that are not yet fully understood. This underscores the need for further research to gain deeper insights into these interactions [41].

## Conclusion

This exploratory study compared pre-selected instrumented gait assessments and identified the gait test with the highest construct validity in PD and APD, to give potential recommendations for assessing gait in the future. As this study followed an exploratory approach, further research is needed to verify the findings of the study and to follow up on questions which arose due to the analysis.

The clinically translatable finding was that the 2MWT tended to show higher associations with clinical, functional, and patient-reported scores compared to the 2 × 10 m test, especially when focusing on the correlations with GV (CV) and SL (CV). These findings from hospital-based 2MWT suggest that valuable information may be provided by non-constant steps, as analysis showed a tendency toward stronger correlations for walking bouts including a higher proportion of acceleration and deceleration steps. This indicates that a separate analysis of constant versus non-constant steps could provide deeper insights into disease-relevant gait patterns. This substudy of the Mobility_APP highlights the need of focusing on a separate analysis of turnings and constant and non-constant steps to investigate their impact on the results. This may hold important information on gait patterns, motor function, and fall risk especially of APD patients. For future studies, developing an algorithm that is able to detect turnings in APD should be a major focus. This study collected extensive gait data in PD and APD, emphasizing the importance of capturing unsupervised physical activity, which goes beyond reflecting results of established scores (construct validity) but provides novel insights into daily life mobility, never investigated before in APD. The analyses of gait assessments indicate potential benefits of exploring long WB, particularly in unsupervised real-world conditions. Measuring gait patterns in daily life introduces a new dimension of gait recordings. Capturing and analyzing intra- and interday variability in the future could provide novel insights for clinicians beyond short hospital visits. Future investigations should focus on underexplored gait features of standardized gait tests in the hospital. Further, PAM should be evaluated in more detail, considering environmental factors and other patient-reported assessments concerning mobility patterns to develop a more comprehensive understanding. As falls are important predictors in PD and APD, including the patient-reported falls efficacy scale [43] may support this understanding in future studies. Additionally, an important next step is to evaluate the walking experience, which may lead to more detailed insights and a better understanding of the real-life data.

## Supplementary Information

Below is the link to the electronic supplementary material.

Supplementary file1 (DOCX 108 KB)

## Data Availability

The data sets analyzed during this study are available from the corresponding authors upon reasonable request.
