# Supplementary material for: Construct validity of instrumented gait assessments in hospital and daily life mobility in patients with Parkinson’s disease and atypical Parkinson's syndromes: an exploratory study
Source: J Neurol. 2026 Feb 9;273(2):122. doi: 10.1007/s00415-026-13652-0 (PMC12883511; doi:10.1007/s00415-026-13652-0)
Supplement: Supplementary file 1 — Supplementary file1 (DOCX 108 KB) [file 415_2026_13652_MOESM1_ESM.docx]

# Supplementary Material

*Table S1 Overview of all scores used for the analysis of the construct validity*

| Category | Scores |
| --- | --- |
| Clinical Scores | Movement Disorder Society-Unified Parkinson's Disease Rating Scale  (MDS-UPDRS Part II + III)  Postural Instability and Gait Difficulty (PIGD) Montreal Cognitive Assessment (MoCA) |
| Functional Scores | Berg Balance Scale (BBS) Timed up and Go (TUG) |
| Patient-reported Scores | Parkinson's Disease Questionnaire (PDQ-8) International Physical Activity Questionnaire (IPAQ) |


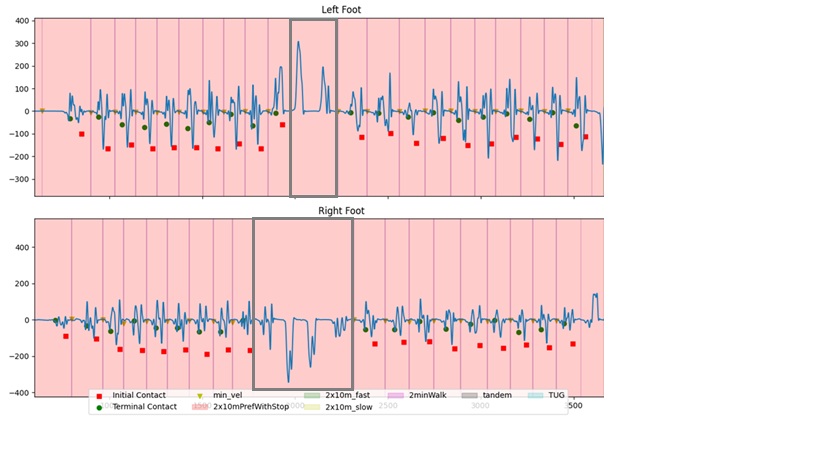


*Figure S2 Example for detection of turning strides for the 2x10m in one patient. The algorithm is not able to detect untypical turning strides, marked in grey. Therefore, all turning strides were excluded for a better comparison.*

Table S3 Spearman correlations for PSP-RS patients to analyze associations between scores and gait parameters - controlled for MoCA.

|  |  | IGA at clinic | | | | | | | | | | physical activity monitoring | | | | | | | | | |
| --- | --- | --- | --- | --- | --- | --- | --- | --- | --- | --- | --- | --- | --- | --- | --- | --- | --- | --- | --- | --- | --- |
|  | Scores | 2x10m | | | | 2 MWT | | | | Short WB | | | | Medium WB | | | | Long WB | | | |
|  |  | GV | SL | GV (CV) | SL (CV) | GV | SL | GV (CV) | SL (CV) | GV | SL | GV (CV) | SL (CV) | GV | SL | GV (CV) | SL (CV) | GV | SL | GV (CV) | SL (CV) |
| PSP-RS | IPAQ MET TOTAL | 0.523 | 0.340 | -0.492 | -0.589 | 0.647 | 0.357 | -0.431 | -0.407 | 0.306 | 0.189 | -0.225 | -0.390 | 0.372 | 0.186 | 0.220 | 0.065 | 0.359 | 0.207 | -0.207 | -0.149 |
|  | PDQ | 0.011 | 0.045 | 0.287 | 0.369 | 0.056 | 0.022 | 0.424 | 0.347 | 0.340 | 0.191 | -0.053 | -0.034 | -0.003 | 0.052 | 0.089 | 0.075 | 0.086 | 0.203 | -0.018 | 0.000 |
|  | MDS-UPDRS II | -0.401 | -0.260 | 0.497 | 0.590 | -0.475 | -0.354 | 0.771 | 0.695 | -0.122 | -0.035 | 0.519 | 0.437 | -0.218 | -0.089 | -0.284 | -0.019 | -0.362 | -0.185 | 0.339 | 0.364 |
|  | TUG | -0.620 | -0.604 | 0.524 | 0.618 | -0.643 | -0.644 | 0.871 | 0.845 | -0.296 | -0.335 | 0.252 | 0.339 | -0.435 | -0.388 | -0.169 | 0.078 | -0.448 | -0.359 | 0.254 | 0.325 |
|  | BBS | 0.646 | 0.676 | -0.459 | -0.516 | 0.714 | 0.719 | -0.749 | -0.745 | 0.421 | 0.353 | -0.306 | -0.179 | 0.572 | 0.510 | 0.057 | -0.195 | 0.461 | 0.428 | -0.248 | -0.307 |
|  | MDS-UPDRS III | -0.559 | -0.696 | -0.003 | 0.170 | -0.741 | -0.806 | 0.544 | 0.577 | -0.543 | -0.514 | 0.169 | 0.245 | -0.571 | -0.539 | -0.153 | 0.148 | -0.667 | -0.609 | 0.106 | 0.184 |
|  | PIGD | -0.661 | -0.685 | 0.328 | 0.483 | -0.755 | -0.715 | 0.603 | 0.616 | -0.541 | -0.430 | 0.284 | 0.250 | -0.419 | -0.365 | -0.143 | 0.066 | -0.461 | -0.426 | 0.317 | 0.370 |

Overview of correlations with MoCA as a confounder compared to unadjusted correlations. Yellow = r decreases from strong (r>.500) to moderate (r<.500)/ moderate to weak (r<.300); light green = r increases from weak to moderate/ moderate to strong; dark green = r increases from weak to strong correlation

*Table S4: Spearman correlations for the three subcohorts to analyze associations between scores and additional gait parameters*

|  |  | **2x10m** | | | | | | **2MWT** | | | | | | |
| --- | --- | --- | --- | --- | --- | --- | --- | --- | --- | --- | --- | --- | --- | --- |
|  |  | Stance time | Stride time | Swing time | Stance time CV | Stride time CV | Swing time CV | Stance time | Stride time | Swing time | Stance time CV | Stride time CV | Swing time CV |  |
| MSA-P | IPAQ MET TOTAL | -0,027 | 0,088 | 0,289 | 0,033 | -0,005 | -0,400 | -0,296 | -0,191 | 0,085 | -0,143 | -0,163 | -0,578 |  |
|  | PDQ | 0,386 | 0,404 | 0,235 | 0,375 | 0,112 | 0,189 | 0,311 | 0,318 | 0,036 | 0,403 | 0,433 | 0,275 |  |
|  | MDS-UPDRS II | 0,191 | 0,145 | -0,006 | 0,349 | 0,234 | 0,439 | 0,159 | 0,079 | -0,099 | 0,372 | 0,447 | 0,516 |  |
|  | MoCA | -0,093 | -0,036 | 0,142 | -0,180 | -0,102 | -0,053 | -0,284 | -0,244 | 0,189 | -0,386 | -0,403 | -0,340 |  |
|  | TUG | 0,196 | 0,096 | -0,229 | 0,167 | 0,171 | 0,386 | 0,367 | 0,266 | -0,110 | 0,268 | 0,353 | 0,562 |  |
|  | BBS | -0,202 | -0,033 | 0,328 | -0,344 | -0,182 | -0,499 | -0,327 | -0,134 | 0,401 | -0,420 | -0,431 | -0,655 |  |
|  | MDS-UPDRS III | 0,103 | 0,103 | -0,114 | 0,173 | 0,080 | 0,361 | 0,045 | 0,061 | -0,078 | 0,102 | 0,134 | 0,334 |  |
|  | PIGD | 0,179 | 0,117 | -0,162 | 0,140 | 0,101 | 0,555 | 0,210 | 0,163 | -0,050 | 0,246 | 0,283 | 0,564 |  |
| PSP-RS | IPAQ MET TOTAL | -0,163 | -0,096 | 0,162 | -0,059 | -0,112 | -0,325 | -0,260 | -0,070 | 0,186 | -0,145 | -0,118 | -0,212 |  |
|  | PDQ | -0,016 | 0,021 | -0,054 | 0,270 | 0,241 | 0,287 | 0,100 | 0,025 | -0,067 | 0,382 | 0,380 | 0,226 |  |
|  | MDS-UPDRS II | 0,133 | 0,234 | 0,249 | 0,329 | 0,309 | 0,512 | 0,356 | 0,302 | 0,178 | 0,521 | 0,505 | 0,404 |  |
|  | MoCA | 0,003 | 0,046 | 0,112 | 0,018 | 0,093 | 0,117 | -0,143 | -0,067 | 0,033 | 0,027 | 0,109 | 0,153 |  |
|  | TUG | 0,546 | 0,476 | 0,001 | 0,430 | 0,331 | 0,545 | 0,486 | 0,398 | 0,031 | 0,310 | 0,292 | 0,594 |  |
|  | BBS | -0,247 | -0,225 | 0,049 | -0,309 | -0,144 | -0,466 | -0,307 | -0,210 | 0,066 | -0,309 | -0,238 | -0,453 |  |
|  | MDS-UPDRS III | 0,243 | 0,272 | 0,010 | -0,034 | -0,111 | 0,294 | 0,423 | 0,290 | -0,030 | 0,360 | 0,416 | 0,536 |  |
|  | PIGD | 0,309 | 0,274 | -0,024 | 0,172 | 0,089 | 0,500 | 0,466 | 0,355 | 0,027 | 0,444 | 0,453 | 0,715 |  |
| PD | IPAQ MET TOTAL | -0,437 | -0,427 | -0,260 | 0,235 | 0,224 | -0,176 | -0,257 | -0,233 | -0,182 | -0,174 | -0,105 | -0,139 |  |
|  | PDQ | 0,349 | 0,311 | 0,098 | -0,114 | -0,050 | 0,242 | 0,072 | 0,034 | -0,043 | -0,105 | 0,006 | 0,230 |  |
|  | MDS-UPDRS II | 0,302 | 0,305 | 0,226 | -0,088 | -0,010 | 0,248 | 0,013 | 0,002 | 0,023 | 0,044 | 0,205 | 0,183 |  |
|  | MoCA | -0,150 | -0,091 | 0,062 | -0,190 | -0,181 | -0,287 | -0,276 | -0,244 | -0,166 | -0,209 | -0,198 | -0,276 |  |
|  | TUG | 0,352 | 0,304 | 0,047 | 0,289 | 0,314 | 0,492 | 0,258 | 0,218 | 0,153 | 0,121 | 0,210 | 0,176 |  |
|  | BBS | -0,395 | -0,344 | -0,001 | -0,051 | -0,114 | -0,325 | -0,118 | -0,049 | 0,093 | -0,201 | -0,283 | -0,293 |  |
|  | MDS-UPDRS III | 0,395 | 0,352 | 0,099 | 0,134 | 0,224 | 0,333 | 0,304 | 0,248 | 0,159 | 0,269 | 0,360 | 0,197 |  |
|  | PIGD | 0,440 | 0,448 | 0,247 | 0,093 | 0,244 | 0,320 | 0,341 | 0,322 | 0,257 | 0,324 | 0,441 | 0,428 |  |

*IPAQ: International Physical Activity Questionnaire; PDQ-8: Parkinson’s Disease Questionnaire; MDS-UPDRS: Movement disorder society Unified Parkinson’s disease rating scale; MoCA: Montreal Cognitive Assessment; TUG: Timed up and go test; BBS: Berg Balance Scale; PIGD: Postural instability and gait difficulty*

*Legend: p > 0.05. and r < .300; p > 0.05 and r > .300; p < 0.05 and r < .500; p < 0.05 and r > .500*
